# Optimization Design of the Bending-Vibration Resistance of Magnetorheological Elastomer Carbon Fibre Reinforced Polymer Sandwich Sheets

**DOI:** 10.3390/ma16062349

**Published:** 2023-03-15

**Authors:** Guangbin Wang, Yangyang Yan, Wenyu Wang, Zelin Li, Zhengwei Zhang, Zhanbin Sun, Zhou Qiao, Jinan Li, Hui Li

**Affiliations:** 1 School of Mechanical and Electrical Engineering, Lingnan Normal University, Zhanjiang 524048, China; 2 Facility Horticulture Laboratory of Universities in Shandong, Weifang University of Science and Technology, Weifang 262700, China; 3 School of Mechanical Engineering and Automation, Northeastern University, Shenyang 110819, China

**Keywords:** multi-objective optimization, optimization design, bending-vibration resistance, composite sheet, magnetorheological elastomer core

## Abstract

An optimization design of the bending-vibration resistance of magnetorheological elastomer carbon fibre reinforced polymer sandwich sheets (MECFRPSSs) was studied in this paper. Initially, by adopting the classical laminate theory, the Reddy’s high-order shear deformation theory, the Rayleigh-Ritz method, etc., an analytical model of the MECFRPSSs was established to predict both bending and vibration parameters, with the three-point bending forces and a pulse load being considered separately. After the validation of the model was completed, the optimization design work of the MECFRPSSs was conducted based on an optimization model developed, in which the thickness, modulus, and density ratios of magnetorheological elastomer core to carbon fibre reinforced polymer were taken as design variables, and static bending stiffness, the averaged damping, and dynamic stiffness parameters were chosen as objective functions. Subsequently, an artificial bee colony algorithm was adopted to execute single-objective, dual-objective, and multi-objective optimizations to obtain the optimal design parameters of such structures, with the convergence effectiveness being examined in a validation example. It was found that it was hard to improve the bending, damping, and dynamic stiffness behaviours of the structure simultaneously as the values of design variables increased. Some compromised results of design parameters need to be determined, which are based on Pareto-optimal solutions. In further engineering application of the MECFRPSSs, it is suggested to use the corresponding design parameters related to a turning point to better exert their bending-vibration resistance.

## 1. Introduction

Carbon fibre reinforced polymer (CFRP) sheets are commonly employed as fundamental mechanical components in the aerospace, ship, and other industries [1,2,3]. They can be easily led by large structural deformation [4], violent vibration [5], and delamination damage [6], due to the frequent service in harsh static and dynamic harsh load environments. Magnetorheological elastomer (MRE) is a new smart material that has a good controllable capability of stiffness and damping effect [7], which can be utilized to improve the mechanical properties of magnetorheological elastomer carbon fibre reinforced polymer sandwich sheets (MECFRPSSs). Based on our previous studies, several efforts were devoted to modelling and analysing the static and dynamic properties of composite sandwich sheet structures with local or overall MRE material or core [8,9,10]. However, it is still a question of how to obtain optimal design results for such structures with better bending-vibration resistance.

Over the past two decades, extensive studies were conducted on static or dynamic resistance of the CFRP sandwich plate structures with MRE cores or other soft materials. However, static and dynamic issues are often investigated separately. For instance, by using an oscillatory rheometry technique, Sun et al. [11] studied the relationship between the magnetic field and the complex shear modulus of MRE materials in the pre-yield regime. Based upon the experimental and numerical results, Ramesh et al. [12] and Aguib et al. [13] evaluated the dynamic behaviours of different MRE sandwich structures under an external magnetic field. They proved that the loss factors of MRE sandwich structure could be increased with increasing the applied magnetic field energy. Babu and Vasudevan [14] performed numerical simulations and experimental tests to estimate the effects of the magnetic field, taper angle of the top and bottom layers, aspect ratio, ply orientations, and various end conditions on the dynamic properties of tapered laminated composite MRE sandwich plates. Kozlowska et al. [15] experimentally investigated the free vibration responses of the CFRP/MRE adaptive beams at different fixed magnetic field levels. They found that the vibration amplitudes of such beams were effectively reduced when the non-homogeneous magnetic field was applied. Aguib et al. [16] and Settet et al. [17] proposed an analytical model of an MRE sandwich beam to predict the static behaviour. They highlighted that the bending resistance decreased as the magnetic field increased. By adopting different experimental methods, Eloy et al. [18,19] examined the dynamic behaviours of the MRE sandwich structures subjected to free and forced vibrations. They found that the natural frequencies and response amplitudes were reduced due to the increase in magnetic field energy. Based on the modified Fourier–Ritz method, Zhang et al. [20] developed an analytical model of porous functionally graded sandwich plates with a viscoelastic core, which had a good prediction capability of vibration and damping properties. Taking the transverse shear in sandwich plates into account, Garbowski et al. [21,22] presented both analytical and experimental investigations into the bending property of sandwich panels with a corrugated core. They found that the transverse shear effect had a big influence on the mechanical behaviour of such panels. Based on the strain energy equivalence, Staszak et al. [23] proposed a shell-to-beam homogenization method to determine the equivalent stiffness of a beam with different cross sections or holes along the length direction.

For the past few years, many optimization design studies were conducted on the bending resistance of composite sandwich sheets regardless of whether the MRE core or other smart material was embedded or not. For example, Theulen and Peijs [24] conducted the optimization design of the bending stiffness and strength of composite sandwich panels with a foam core and verified the optimization results with the four-point bending test data. They used a 2D sandwich model of truss-cored sandwich plates subjected to bending, transverse shear, and in-plane compression loads, Liu et al. [25] proposed an optimization method to determine the minimum structural weights with various failure constraints. Li et al. [26] developed a minimum weight optimization method for composite sandwich sheets subjected to combined torsion and bending loads. Based on a two-level optimisation strategy, Catapano and Montemurro [27] proposed a novel least-weight optimum design method for honeycomb sandwich panels, which could maintain a good bending stiffness of the panel. Hao et al. [28] performed a strength-to-weight optimization strategy for wood-based sandwich sheets with a paper honeycomb core. They also validated the optimization results using a quasi-static three-point bending test method. Using the finite element method and multi-objective optimization of design variables, Uzay et al. [29] proposed an optimal design method for the CFRP sheets with a polymer foam core to obtain the optimal solutions of structural mass and bending bearing capacity.

The optimization design studies on vibration resistance of various composite sandwich sheets with and without cores were also reported. For example, using the genetic algorithm and simulated annealing approach, Karakaya and Soykasap [30] performed an optimization design of the stacking sequence for maximizing natural frequencies and buckling resistance of laminated hybrid composite plates. Based on the genetic algorithm and the layer-wise optimization concept, Honda and Narita [31] investigated a single optimization issue of composite sheets with a locally anisotropic structure, where the maximized fundamental frequency was taken as the object function and fibre shapes were design variables. For minimizing weight and material cost and maximizing modal damping, Madeira et al. [32] carried out a multi-objective optimization analysis of viscoelastic-laminated composite sandwich sheets. Additionally, the trade-off Pareto-optimal solutions were discussed in detail. Based on an analytical model of sandwich panels with a multi-cellular core, Alfouneh et al. [33] studied the multi-objective optimal design of the core to minimize the structural dynamic response under harmonic excitation. Wang et al. [34] proposed a high-efficiency optimization procedure to obtain the optimal structural stiffness and vibration attenuation of laser-welded corrugated-core sandwich panels with polyurea-metal skins. By combing the response surface optimization method with finite element models established in ANSYS software, Njim et al. [35] conducted the optimization design of functionally graded (FG) porous sandwich sheets, in which the maximized fundamental frequencies and the minimized mass of the FG core were considered.

To fulfil the increasing demand of engineering industry, both the bending and vibration-resistant capabilities of composite sandwich sheets need to be considered. However, rare optimization design work was carried out on bending-vibration resistance. Thankfully, considerable studies [36,37,38,39,40] have reported on the research progress on the vibration and bending characteristics of composite structures. For instance, based on a finite element model with a nine-noded plate element, Tu and Quoc [36] performed the bending and free vibration analysis of composite and sandwich laminate sheets. By using an edge-based smoothing approach, Cui et al. [37] estimated the natural frequencies and bending deformation of composite sheets. Natarajan and Manickam [38] explored the static bending and free vibration behaviours of functionally graded material sheets based on the proposed QUAD-8 shear flexible element to consider the realistic variation of displacements along the thickness direction. To predict and evaluate the vibration and bending responses of double-core sandwich panels with a harmonic patch pressure load, Kapuria and Nath [39] created a new zigzag local theory with nine primary variables. Khiloun et al. [40] presented a new high-order shear and normal deformation theory for the static bending and free vibration analysis of FG sheets. Additionally, they used a number of numerical examples to verify the accuracy of their theory. 

According to the literature review presented in this study, no research work was undertaken on the optimal design of the bending-vibration resistance of composite sandwich sheets with an MRE core. To cover this research gap, a theoretical model of the MECFRPSSs is established in Section 2, which can be utilized to predict both bending and vibration parameters. Then, an optimization model of the MECFRPSSs is developed in Section 3 and an artificial bee colony algorithm is adopted to execute single-objective, dual-objective, and multi-objective optimizations to obtain the optimal design parameters in Section 4. The current study offers an effective route for improving the bending-vibration resistant performance of the MECFRPSSs, which will help in promoting the application of composite MRE sandwich structures in a wide range of engineering sectors.

## 2. Analysis of Bending and Vibration Resistances

### 2.1. Model Description

**Figure 1:**
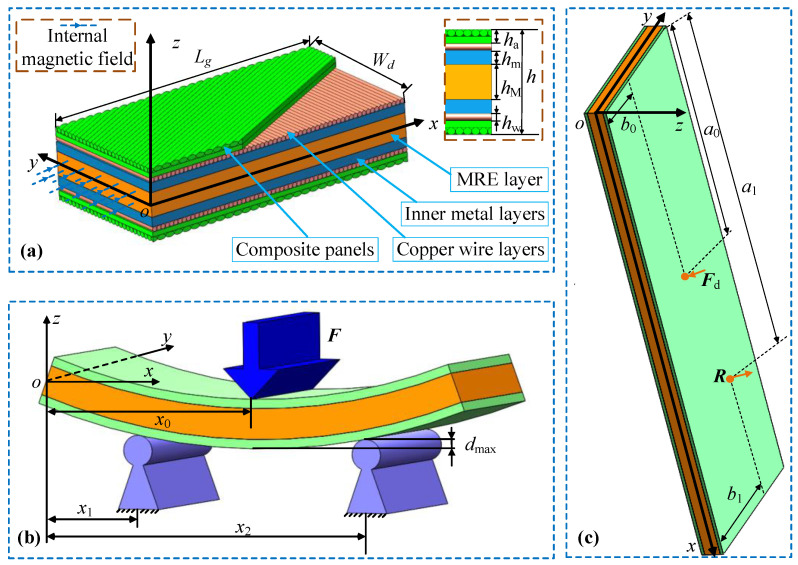
A model for analysis of bending and vibration parameters of the MECFRPSSs: (**a**) coordinate and dimension, (**b**) deformation with three-point bending forces, and (**c**) dynamic response with a pulse load.

A rectangle MECFRPSS with top and bottom composite panels and an MRE core (MREC) was investigated, as illustrated in Figure 1a. The MREC contains two copper wire layers, two inner metal layers, and one MRE layer. Here, an overall coordinate *o*-*xyz* was supposed to be prepared at the mid-plane of the MREC. *L_g_*, *h*, and *W_d_* represent the overall length, thickness, and width of the structure, respectively, and *h*_a_, *h*_w_, *h*_m_, and *h*_M_ denote the thickness of the panels, copper wire layers, two inner metal layers, and MRE layer. To analyse its bending behaviour, the three-point bending forces were assumed to be applied on the top and bottom panels, as demonstrated in Figure 1b, where a concentrated line force *F* was applied to the upper panel with maximum deformation *d*_max_, and *x*_0_, *x*_1_, and *x*_2_ were the coordinates of the line force and two support reaction forces about the *x*-axis of the structure. In addition, to analyse the vibration parameters of the MECFRPSS structure, at the excitation point *F*_d_(*a*_0_, *b*_0_), a pulse load was assumed to be applied on the structure, and the relevant dynamic response was located at the point *R*(*a*_1_, *b*_1_), as displayed in Figure 1c.

In addition, the following assumptions were adopted in the modelling process:

(1) There is no slippage between the layers of the MECFRPSS structure since each layer is securely bound;
(2) The internal magnetic field only affects the MRE materials and the magnetic field effect in the *z* direction is ignored because thin copper wire layers are adopted in the MECFRPSS structure;
(3) The heating effect of the magnetic field is ignored because the related current is small;
(4) The bending deflection of the MECFRPSS structure is supposed to be elastic, i.e., the structure can be recovered as the bending force is removed;
(5) When the bending problem is solved, the change of material parameters of MRE affected by the inside magnetic field is ignored due to its weak influence on static bearing stiffness.

### 2.2. Material Properties of MRE Core

By combining the complex modulus method [41,42] with Jolly theory [43], the equivalent moduli $E_{C1}^{*}$, $E_{C2}^{*}$, $G_{C12}^{*}$, and $G_{C23}^{*}$ of the MRE materials can be considered as a function of magnetic induction intensity *M*_a_, which is generated by the copper coils in the MECFRPSS structure. As a result, $E_{C1}^{*}$, $E_{C2}^{*}$, $G_{C12}^{*}$, and $G_{C23}^{*}$ can be defined as:

(1a) $$E_{C1}^{*}=(E_{C1}+d_{1}M_{a}{}^{e_{1}})(1+i\eta_{C1}f_{1}M_{a}{}^{g_{1}})$$

(1b) $$E_{C2}^{*}=(E_{C2}+d_{2}M_{a}{}^{e_{2}})(1+i\eta_{C2}f_{2}M_{a}{}^{g_{2}})$$

(1c) $$G_{C12}^{*}=\left[G_{C12}+d_{3}M_{a}{}^{e_{3}}(v_{c}p_{c}\chi_{0}^{2}/2p_{m}p_{v}^{2}g_{c}^{3})\right]\left[1+i\eta_{C12}f_{3}M_{a}{}^{g_{3}}(v_{c}p_{c}\chi_{0}^{2}/2p_{m}p_{v}^{2}g_{c}^{3})\right]$$

(1d) $$G_{C23}^{*}=(G_{C23}+d_{4}M_{a}{}^{e_{4}})(1+i\eta_{C23}f_{4}M_{a}{}^{g_{4}})$$

where $E_{C1}$, $E_{C2}$, *G*_C12_, and *G*_C23_ are the normal elastic moduli of MRE when the magnetorheological effect is ignored; $\eta_{C1}$, $\eta_{C2}$, $\eta_{C12}$, and $\eta_{C23}$ are the corresponding loss factors; i is the imaginary unit; *d_i_*, *e_i_*, *f_i_*_,_ and *g_i_* (*i* = 1, 2, 3, 4) are the magnetic field coefficients that can be determined on the basis of the tested frequency response function (FRF) data under different magnetic levels [44]; *v*_c_ is the volume fraction of carbonyl iron particles (CIPs); *p*_v_, *p*_c_, and *p*_m_ represent the permeabilities of the vacuum, CIPs and MRE, respectively; *g*_c_ is the mutual gap in the CIPs. *v*_c_ *p*_v_, *p*_c_ *p*_m_, and *g*_c_ are the related parameters when the magnetorheological effect is considered [45].

### 2.3. Analysis of Bending Resistance

To solve the bending stiffness of the MECFRPSS structure, the longitudinal, transverse and shear moduli of each layer should be treated equivalently, which means that it is necessary to consider the deformations of constituted layers or parts of the structure studied in the main directions in the *o*-*xyz* coordinate system. To achieve this purpose, the equivalent Young’s moduli of composite panels ${\bar{E}}_{f}$, copper wire layers ${\bar{E}}_{w}$, inner metal layers ${\bar{E}}_{\mathrm{IM}}$, and MRE layer ${\bar{E}}_{C}$ in the *x* direction are firstly defined as:

(2) $$\begin{array}{c} {\bar{E}}_{f}=12\sum_{k=1}^{n_{f}}\int_{h_{k}^{f}}\left[Q_{11}^{f}\cos^{4}\theta^{k}+Q_{22}^{f}\sin^{4}\theta^{k}+2(Q_{12}^{f}+2Q_{66}^{f})\sin^{2}\theta^{k}\cos^{2}\theta^{k}\right]z^{2}dz/h_{f}^{3}\\ {\bar{E}}_{w}=12\sum_{k=1}^{n_{w}}\int_{h_{k}^{w}}\left[Q_{11}^{w}\cos^{4}\theta^{k}+Q_{22}^{w}\sin^{4}\theta^{k}+2(Q_{12}^{w}+2Q_{66}^{w})\sin^{2}\theta^{k}\cos^{2}\theta^{k}\right]z^{2}dz/h_{w}^{3}\\ {\bar{E}}_{\mathrm{IM}}=E_{\mathrm{IM}}\\ {\bar{E}}_{C}=E_{C1} \end{array}$$

where $n_{f}$ and $n_{w}$ are the total layer number of the panels and copper wire layers, respectively; $h_{k}^{f}$ and $h_{k}^{w}$ are the thicknesses of the *k*-th layer of the panels and copper wire layers, respectively; $\theta^{k}$ is the angle between the directions of the *x*-axis and *k*-th layer in the panels; $E_{\mathrm{IM}}$ is the Young’s modulus of inner metal layers; $Q_{ij}^{f}$ and $Q_{ij}^{w}$ (*i*, *j* = 1, 2, 6) represents the reduced stiffness coefficients of the panels and copper wire layers, respectively, with the detailed expressions being [46]:

(3a) $$\left[Q_{11}^{f},Q_{12}^{f},Q_{22}^{f},Q_{66}^{f}\right]=\left[\frac{E_{1}^{f}}{1-\upsilon_{12}^{f}\upsilon_{21}^{f}},\frac{\upsilon_{12}^{f}E_{1}^{f}}{1-\upsilon_{12}^{f}\upsilon_{21}^{f}},\frac{E_{2}^{f}}{1-\upsilon_{12}^{f}\upsilon_{21}^{f}},G_{12}^{f}\right]$$

(3b) $$\left[Q_{11}^{w},Q_{12}^{w},Q_{22}^{w},Q_{66}^{w}\right]=\left[\frac{E_{1}^{w}}{1-\upsilon_{12}^{w}\upsilon_{21}^{w}},\frac{\upsilon_{12}^{w}E_{1}^{w}}{1-\upsilon_{12}^{w}\upsilon_{21}^{w}},\frac{E_{2}^{w}}{1-\upsilon_{12}^{w}\upsilon_{21}^{w}},G_{12}^{w}\right]$$

where $E_{1}^{f}$ and $E_{1}^{w}$ are the longitudinal Young’s moduli of the composite panels and copper wire layer, respectively; $E_{2}^{f}$ and $E_{2}^{w}$ are the corresponding transverse Young’s moduli; $G_{12}^{f}$ and $G_{12}^{w}$ are the corresponding shear moduli; $v_{12}^{f}$, $v_{21}^{f}$, $v_{12}^{w}$, and $v_{21}^{w}$ are the corresponding Poisson’s ratios, respectively.

The bending stiffness parameter *D*_P_ of the MECFRPSSs can be derived as [47]:

(4) $$D_{P}={\bar{E}}_{f}\left\{h^{3}-{\left[2(h_{w}+h_{m})+h_{M}\right]}^{3}\right\}W_{d}/12+D_{C}$$

where *D*_C_ is the bending stiffness of the MREC, which has the following form:

(5) $$D_{C}=\frac{W_{d}}{12}\left\{{\bar{E}}_{w}{\left[2(h_{w}+h_{m})+h_{M}\right]}^{3}+({\bar{E}}_{\mathrm{IM}}-{\bar{E}}_{w}){\left(h_{M}+2h_{m}\right)}^{3}+({\bar{E}}_{C}-{\bar{E}}_{\mathrm{IM}})h_{M}^{3}\right\}$$

Since the MRE material has a low shear stiffness behaviour, its deformation effect must be taken into account. Hence, the shear stiffness *S*_C_ of MRE layer in the MECFRPSS structure can be defined as:

(6) $$S_{C}=G_{C12}W_{d}h_{M}$$

Furthermore, the maximum deformation *d*_max_ of the structure subjected to three-point bending forces is determined as:

(7) $$d_{\max}=d_{b}+d_{s}=\frac{F{\left(x_{2}-x_{1}\right)}^{3}}{12D_{P}}\left[\frac{3}{4}\left(\frac{x_{0}-x_{1}}{x_{2}-x_{1}}\right)-{\left(\frac{x_{0}-x_{1}}{x_{2}-x_{1}}\right)}^{3}\right]+\frac{F\left(x_{0}-x_{1}\right)}{2S_{C}}$$

where *d*_s_ and *d*_b_ are the deformations related to shear and bending forces, respectively.

The shear stiffness *K*_s_ and the bearing stiffness *K*_b_ of the MECFRPSS structure are stated as:

(8a) $$K_{s}=\frac{F}{d_{s}}=\frac{2S_{C}}{x_{0}-x_{1}}$$

(8b) $$K_{b}=\frac{F}{d_{b}}=\frac{12D_{P}}{\left[3(x_{0}-x_{1}){\left(x_{2}-x_{1}\right)}^{2}/4-{\left(x_{0}-x_{1}\right)}^{3}\right]}$$

Finally, the static bending stiffness $\bar{K}$ of the MECFRPSSs subjected to the three-point bending forces can be obtained as:

(9) $$\bar{K}=\frac{F}{d_{b}+d_{s}}=\frac{K_{b}K_{s}}{K_{b}+K_{s}}=1/\left[\frac{{\left(x_{2}-x_{1}\right)}^{3}}{12D_{P}}\left[\frac{3}{4}\left(\frac{x_{0}-x_{1}}{x_{2}-x_{1}}\right)-{\left(\frac{x_{0}-x_{1}}{x_{2}-x_{1}}\right)}^{3}\right]+\frac{x_{0}-x_{1}}{2S_{C}}\right]$$

### 2.4. Analysis of Vibration Resistance

To obtain the vibration solution of the MECFRPSS structure with a high accuracy, the classical laminate theory [48] (applied to the top and bottom panels) and the Reddy’s high-order shear deformation theory [49] (applied to the MREC) were adopted in this paper. As a result, the displacement field functions of the structure studied can be given as:

(10a) $$\left[u_{c},v_{c},w_{c}\right]=\left[u_{0}-z\frac{\partial w}{\partial x},v_{0}-z\frac{\partial w}{\partial y},w_{0}\right]$$

(10b) $$\left[u_{h},v_{h},w_{h}\right]=\left[u_{0}+z\psi_{x}-\frac{4z^{3}}{3h^{2}}\left(\frac{\partial w}{\partial x}+\psi_{x}\right),v_{0}+z\psi_{y}-\frac{4z^{3}}{3h^{2}}\left(\frac{\partial w}{\partial y}+\psi_{y}\right),w_{0}\right]$$

where *u*_c_, *v*_c_, and *w*_c_ are the displacement components of the panels; *u*_h_, *v*_h_, and *w*_h_ are the displacement components of the MREC; *u*_0_, *v*_0_, and *w*_0_ are the displacement components of the mid-plane of the structure along with the *x*, *y*, and *z* directions, respectively; $\psi_{x}$ and $\psi_{y}$ are the transverse normal rotation variables in the *xoz* and *yoz* planes.

According to the Rayleigh–Ritz approach [50], *u*_0_, *v*_0_, *w*_0_, $\psi_{x}$, and $\psi_{y}$ of the MECFRPSS are assumed as:

(11) $$\begin{array}{l} u_{0}=e^{i\omega t}\sum_{m=1}^{M}\sum_{n=1}^{N}A_{mn}P_{m}(\xi )P_{n}(\eta )\\ v_{0}=e^{i\omega t}\sum_{m=1}^{M}\sum_{n=1}^{N}B_{mn}P_{m}(\xi )P_{n}(\eta )\\ w_{0}=e^{i\omega t}\sum_{m=1}^{M}\sum_{n=1}^{N}C_{mn}P_{m}(\xi )P_{n}(\eta )\\ \psi_{x}=e^{i\omega t}\sum_{m=1}^{M}\sum_{n=1}^{N}D_{mn}P_{m}(\xi )P_{n}(\eta )\\ \psi_{y}=e^{i\omega t}\sum_{m=1}^{M}\sum_{n=1}^{N}E_{mn}P_{m}(\xi )P_{n}(\eta ) \end{array}$$

where *ω* is the excitation frequency when the pulse load is applied on the structure; $A_{mn},\;B_{mn},\;C_{mn},\;D_{mn},\;E_{mn}$ (m=1,…,M; n=1,…,N) are the Ritz vectors; *N* and *M* are the truncation values; and *P_m_*(*ξ*) and *P_n_*(*η*) are the orthogonal polynomials which can be determined based on the selected boundary constraints [51].

The kinetic energy *T*_C_ and strain energy *U*_C_ of the MREC are, respectively, determined as:

(12a) $$\begin{array}{c} T_{C}=\rho_{w}\int_{A}\int_{h_{w}}\left({\left(\frac{\partial u_{c}}{\partial t}\right)}^{2}+{\left(\frac{\partial v_{c}}{\partial t}\right)}^{2}+{\left(\frac{\partial w_{c}}{\partial t}\right)}^{2}\right)dzdA+\rho_{\mathrm{IM}}\int_{A}\int_{h_{m}}\left({\left(\frac{\partial u_{c}}{\partial t}\right)}^{2}+{\left(\frac{\partial v_{c}}{\partial t}\right)}^{2}+{\left(\frac{\partial w_{c}}{\partial t}\right)}^{2}\right)dzdA\\ \;+\frac{1}{2}\rho_{M}\int_{A}\int_{h_{M}}\left({\left(\frac{\partial u_{h}}{\partial t}\right)}^{2}+{\left(\frac{\partial v_{h}}{\partial t}\right)}^{2}+{\left(\frac{\partial w_{h}}{\partial t}\right)}^{2}\right)dzdA \end{array}$$

(12b) $$\begin{array}{c} U_{C}=\int_{A}\left[(M_{x}^{w}+M_{x}^{m})\kappa_{x}+(M_{y}^{w}+M_{y}^{m})\kappa_{y}+(M_{xy}^{w}+M_{xy}^{m})\kappa_{xy}\right]dA\\ \;+\frac{1}{2}\int_{A}\int_{-\frac{h_{M}}{2}}^{\frac{h_{M}}{2}}(\sigma_{x}^{c}\varepsilon_{x}^{c}+\sigma_{y}^{c}\varepsilon_{y}^{c}+\sigma_{xy}^{c}\varepsilon_{xy}^{c}+\sigma_{xz}^{c}\varepsilon_{xz}^{c}+\sigma_{yz}^{c}\varepsilon_{yz}^{c})dzdA \end{array}$$

where $\rho_{w}$, $\rho_{\mathrm{IM}}$, and $\rho_{M}$ are the densities of the copper wire layers, inner metal layers, and MRE layer, respectively; $M_{x}^{w}$, $M_{y}^{w}$, and $M_{xy}^{w}$ are the internal moments of the copper wire layers; $M_{x}^{m}$, $M_{y}^{m}$, and $M_{xy}^{m}$ are the internal moments of the inner metal layers; $\kappa_{x}$, $\kappa_{y}$, and $\kappa_{xy}$ are the related curvature coefficients of the MREC; $\sigma_{x}^{c}$ and $\sigma_{y}^{c}$ are the normal stresses in the *x*-axis and *y*-axis, respectively; $\sigma_{xy}^{c}$, $\sigma_{xz}^{c}$, and $\sigma_{yz}^{c}$ are the shear stresses at the *xoy*, *xoz*, *yoz* planes, respectively; $\varepsilon_{x}^{c}$, $\varepsilon_{y}^{c}$, $\varepsilon_{xy}^{c}$, $\varepsilon_{xz}^{c}$, and $\varepsilon_{yz}^{c}$ are the corresponding strains of the structure.

Then, the total kinetic energy *T* and strain energy *U* can be determined as:

(13a) $$T=\rho_{f}\int_{A}\int_{h_{f}}\left[{\left(\frac{\partial u_{c}}{\partial t}\right)}^{2}+{\left(\frac{\partial v_{c}}{\partial t}\right)}^{2}+{\left(\frac{\partial w_{c}}{\partial t}\right)}^{2}\right]dzdA+T_{C}$$

(13b) $$U=\int_{A}\left(M_{x}^{f}\kappa_{x}+M_{y}^{f}\kappa_{y}+M_{xy}^{f}\kappa_{xy}\right)dA+U_{C}$$

where $\rho_{f}$ is the density of the composite panels; $M_{x}^{f}$, $M_{y}^{f}$, and $M_{xy}^{f}$ are the internal moments of the composite panels.

Using the Rayleigh–Ritz approach, the Lagrangian function *L* can be obtained as:

(14) L=U−T

By minimizing *L* with regard to *A_mn_*, *B_mn_*, *C_mn_*, *D_mn_*, and *E_mn_*, one has:

(15) $$\frac{\partial L}{\partial A_{mn}}=\frac{\partial L}{\partial B_{mn}}=\frac{\partial L}{\partial C_{mn}}=\frac{\partial L}{\partial D_{mn}}=\frac{\partial L}{\partial E_{mn}}=0$$

Equation (15) can be written in the following matrix forms:

(16) $$\begin{array}{c} C=\mathrm{diag}\left[\frac{\partial U_{i}}{\partial A_{mn}},\;\frac{\partial U_{i}}{\partial B_{mn}},\;\frac{\partial U_{i}}{\partial C_{mn}},\;\frac{\partial U_{i}}{\partial D_{mn}},\;\frac{\partial U_{i}}{\partial E_{mn}}\right]\\ K=\mathrm{diag}\left[\frac{\partial U_{r}}{\partial A_{mn}},\;\frac{\partial U_{r}}{\partial B_{mn}},\;\frac{\partial U_{r}}{\partial C_{mn}},\;\frac{\partial U_{r}}{\partial D_{mn}},\;\frac{\partial U_{r}}{\partial E_{mn}}\right]\\ M=\mathrm{diag}\left[\frac{\partial T}{\partial A_{mn}},\;\frac{\partial T}{\partial B_{mn}},\;\frac{\partial T}{\partial C_{mn}},\;\frac{\partial T}{\partial D_{mn}},\;\frac{\partial T}{\partial E_{mn}}\right]/\omega_{q}^{2} \end{array}$$

where ***C***, ***K***, and ***M*** are the damping, stiffness, and mass matrices of the MECFRPSS structure; *ω_q_* is the *q*-th natural frequency concerned; $U_{i}$ and $U_{r}$, respectively, are the imaginary and real parts of *U*.

The free vibration equations of the MECFRPSS structure are stated as:

(17) $$(K-\omega_{r}^{2}M)e=0$$

where ***e*** is the eigenvector. After the *q*-th natural frequency and eigenvector are solved, each modal shape can be determined by substituting ***e*** into Equation (11).

To solve the damping ratio of the structure, the dissipated energy Δ*U_q_* and strain energy *U_q_* with the *q*-th mode are determined as:

(18) $$\begin{array}{c} \Delta U_{q}=2\pi e_{q}^{T}Ce_{q}\\ U_{q}=e_{q}^{T}Ke_{q} \end{array}$$

Furthermore, the damping ratio $\xi_{q}$ associated with the *q*-th mode can be solved as:

(19) $$\xi_{q}=\frac{\Delta U_{q}}{4\pi U_{q}}$$

By applying the orthogonality principle of mode shape [52], the following expressions between ***K***, ***C***, and ***M*** can be obtained:

(20) $$\begin{array}{c} K+iC={\left(e^{T}\right)}^{-1}\mathrm{diag}\left(\omega_{q}^{2}(1+i2\xi_{q})\right)e^{-1}\\ M={\left(e^{T}\right)}^{-1}e^{-1} \end{array}$$

Then, the FRF matrix ***H***(*ω*) can be expressed as [53]:

(21) $$H\left(\omega \right)={\left[K+iC-\omega^{2}M\right]}^{-1}={\left[{\left(e^{T}\right)}^{-1}\mathrm{diag}\left(\omega_{q}^{2}(1+i2\xi_{q})-\omega^{2}\right)e^{-1}\right]}^{-1}=\sum_{q=1}^{MN}\frac{e_{q}^{T}e_{q}}{\omega_{q}^{2}\left(1+i2\xi_{q}\right)-\omega^{2}}$$

Finally, the dynamic stiffness *K*_d_ of the MECFRPSS at the point *R*(*a*_1_, *b*_1_) under pulse excitation force can be obtained as:

(22) $$K_{d}(\omega )={\left[H(\omega )\right]}^{-1}={\left[\sum_{q=1}^{MN}\frac{W_{q}\left(a_{0},b_{0}\right)W_{q}\left(a_{1},b_{1}\right)}{\omega_{q}^{2}\left(1+i2\xi_{q}\right)-\omega^{2}}\right]}^{-1}$$

where H(ω) is the FRF of the MECFRPSS structure; *W_q_*(*a*_0_, *b*_0_) and *W_q_*(*a*_1_, *b*_1_) are the *q*-th shape functions with respect to the excitation and response points.

### 2.5. Validation of Theoretical Model

First, the experimental results from Ref. [10] were used to verify the current model in predicting the vibration resistances of the MECFRPSSs. The cantilever boundary conditions of the specimens were achieved by a set of clamping fixtures. The material and geometric parameters of panels, copper wire layers, inner metal layers, and MRE layer were provided by Ref. [10], as shown in Table 1. Note that the magnetic field coefficients were obtained from the FRF data tested under different magnetic levels, as listed in Table 2. Additionally, Table 3 and Table 4 show the comparison results of the dynamic stiffness values and damping ratios obtained by our model and Ref. [10] with different magnetic induction amplitudes. Here, a good agreement was observed, as there were only small relative deviations in the dynamic stiffness and damping ratios with the corresponding maximum values being 4.7 and 9.0 %, respectively, which proves that the proposed model was capable of predicting the vibration resistances of the MECFRPSSs. The above deviations may be caused by: (1) the neglect of the interlayer stress effect between two panels and MREC in the current model; (2) the neglect of the temperature effect of the copper wire due to continuous application of the internal current.

In addition, the finite element (FE) results calculated based on ANSYS workbench software were also utilized to validate our model when the bending deformation of the MECFRPSS structure was predicted. Here, the identical material and geometric parameters listed in Table 1 were adopted with a concentrated line force *F* being set as 20, 30, and 40 N, and *x*_0_ = 0.1 m, *x*_1_ = 0.05 m, *x*_2_ = 0.15 m, respectively. 

**Figure 2:**
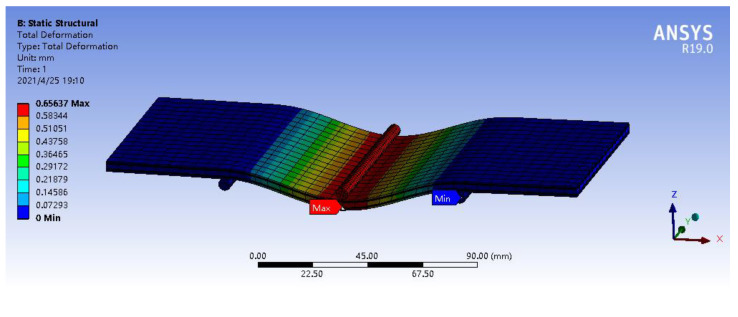
Deformation map of the MECFRPSS structure subjected to three-point bending forces when the concentrated line force was 20N.

By taking the concentrated line force that was equal to 20 N as an example, Figure 2 provides the FE deformation map of the MECFRPSS structure subjected to three-point bending forces, where the maximum deformation value was also extracted for further analysis. After that, Table 5 presents a comparison of the maximum deformations and static bending stiffness values calculated by ANSYS workbench software and the current model with different line forces. A reasonably good agreement between the calculations and measurements was clearly observed, as the related discrepancies in the calculated maximum deformations and static bending stiffness values were less than 4.6 and 4.4 %, respectively. Thus, the current model can keep a good accuracy in the prediction of the bending resistance of the MECFRPSS structure. The above calculation deviations may be from the following factors: (1) the plastic deformation effect was ignored in the present model; (2) the different simulation methods were adopted in the present study and FE software when three-point bending forces were considered to apply to the MECFRPSS structure.

## 3. Optimal Design Formulation

### 3.1. Optimization Model 

The main purpose of the present study was to realize the optimal design of bending-vibration resistance for the MECFRPSSs. Thus, the static bending stiffness, averaged damping, and average dynamic stiffness were considered as the objective functions. 

The first objective was maximizing the static bending stiffness $\bar{K}$ of the MECFRPSSs. Here, to facilitate optimization operation, the maximization problem was further turned into a minimization one by solving its opposite counterpart value. Therefore, the first objective function $\phi_{1}$ was defined as

(23) $$\phi_{1}=-\bar{K}$$

To maximize the averaged damping of the MECFRPSSs, the second objective function $\phi_{2}$ was defined as:

(24) $$\phi_{2}=-\sum_{q=1}^{X}a_{q}\xi_{q}$$

where *X* is the mode order considered, and $a_{q}$ is the *q*-th weighting coefficient for damping ratio.

Considering the objective of maximizing the averaged dynamic stiffness of the MECFRPSSs, the third objective function $\phi_{3}$ is written as:

(25) $$\phi_{3}=-\sum_{q=1}^{N}b_{q}K_{d}^{q}$$

where $K_{d}^{q}$ is the *q*-th dynamic stiffness, and $b_{q}$ is the *q*-th weighting coefficient for dynamic stiffness. 

Moreover, suppose $h_{s}$ is the thickness ratio of MRE to panels with the following expression:

(26) $$h_{s}=h_{M}/h_{a}$$

Assume that $E_{s}$ is the modulus ratio of MRE to panels with the following expression:

(27) $$E_{s}=E_{C1}/E_{1}^{f}$$

Similarly, assume that $\rho_{s}$ is the density ratio of MRE to panels with the following expression:

(28) $$\rho_{s}=\rho_{M}/\rho_{f}$$

Then, $h_{s}$, $E_{s}$, and $\rho_{s}$ are determined as design variables, which need to meet the following constraint conditions:

(29) $$h_{s}{}_{\min}\leq h_{s}\leq h_{s}{}_{\max}$$

(30) $$\;E_{s}{}_{\min}\leq E_{s}\leq E_{s}{}_{\max}$$

(31) $$\rho_{s}{}_{\min}\leq \rho_{s}\leq \rho_{s}{}_{\max}$$

where $h_{s}{}_{\min},\;h_{s}{}_{\max}$, $E_{s}{}_{\min},\;E_{s}{}_{\max}$, and $\rho_{s}{}_{\min},\;\rho_{s}{}_{\max}$ are the upper and lower constraint boundary values for $h_{s}$,$E_{s}$, and $\rho_{s}$ respectively. 

Finally, with combination of Equations (23)–(31), the multi-objective optimization model of the MECFRPSSs can be constructed as:

(32) $$\begin{array}{c} \min\;\phi_{1}\left(h_{s},E_{s},\rho_{s}\right),\;\phi_{2}\left(h_{s},E_{s},\rho_{s}\right),\;\phi_{3}\left(h_{s},E_{s},\rho_{s}\right)\\ s.\;t.\;\;\;\;\;h_{s}{}_{\min}\leq h_{s}\leq h_{s}{}_{\max}\\ E_{s}{}_{\min}\leq E_{s}\leq E_{s}{}_{\max}\\ \rho_{s}{}_{\min}\leq \rho_{s}\leq \rho_{s}{}_{\max} \end{array}$$

### 3.2. Optimization Algorithm

The artificial bee colony (ABC) algorithm [54,55] was as simple and flexible as genetic algorithms, differential evolution, evolutionary strategy, and particle swarm optimization algorithms. However, the ABC algorithm employed fewer control parameters. Thus, this algorithm was used in this work to execute the optimization design owing to the fast convergence and high accuracy. Assume that XP is the initial population vector and *NP* is the population size of employed bees, and XP has the following expression:

(33) $$XP=\left[xp_{1},\;xp_{2},\;\ldots \;,xp_{D}\;\right]$$

where $xp_{i}$(i=1, 2,…, D) is the *i*-th nectar position. Note that in the ABC algorithm, the nectar position and employed bee corresponded to each other. 

In this way, if the $t_{r}$-th iteration is conducted smoothly, the *i*-th nectar position vector $XP^{t_{r}}$ after this iteration can be obtained as:

(34) $$XP^{t_{r}}=\left[xp_{1}^{t_{r}},\;xp_{2}^{t_{r}},\;\ldots \;,xp_{D}^{t_{r}}\;\right]$$

where $xp_{i}^{t_{r}}$(i=1, 2,…, D) is the *i*-th nectar position via the $t_{r}$-th iteration.

In Equation (34), the corresponding initialization value of $xp_{i}^{t_{r}}$ is formulated as:

(35) $$xp_{i}^{t_{r}}=L_{i}+\mathrm{rand}\left(0,1\right)\left(U_{i}-L_{i}\right)$$

where $L_{i}$ and $U_{i}$ represent the up and low limits of search space of employed bees, respectively. 

Furthermore, the employed bees will update the nectar positions. When the *i*-th nectar position is updated, one has:

(36) $$vp_{i}=xp_{i}+\varphi \left(xp_{i}-xp_{j}\right)a_{c}$$

where j=1, 2,…, NP(j≠i) represents a number that is randomly selected among all of nectar sources but it should not be equal to i, and φ is the random number uniformly distributed in the range of [−1, 1], which can be determined by disturbance amplitude in the ABC algorithm [55], $a_{c}$ is acceleration coefficient with the expressions being provided in Ref. [56]. 

When the fitness value of new nectar position vector $VP=\left[vp_{1},\;vp_{2},\;\ldots \;,vp_{D}\;\right]$ is better than the one of XP, the Greedy evolutionary law (GEL) [55] is adopted to replace XP with VP. Otherwise, XP is reserved in the search process of nectar source. 

Then, after all employed bees complete the corresponding nectar location search in Equation (36), they fly back to the information exchange area to share nectar position information. According to iteration principle of the ABC algorithm [55], the onlooker bees will follow them with the probability function $P_{i}$, which can be defined as:

(37) $$P_{i}=fit_{i}/\sum_{i=1}^{NP}fit_{i}$$

where $fit_{i}$ is the fitness evaluation function. Assuming that $\phi (xp_{i})$ is the related objective function value of $xp_{i}$, then $fit_{i}$ in the ABC algorithm can be expressed as

(38) $$fit_{i}=\left\{ \begin{array}{l} 1/[1+\phi (xp_{i})]\;\;\;\;\;\phi (xp_{i})>0\\ 1+\left|\phi (xp_{i})\right|\;\;\;\;\;\;\;\;\phi (xp_{i})\leq 0 \end{array}\right.$$

Following that, each onlooker bee adopts a roulette method to choose the corresponding employed bee, which means a uniformly distributed random number will be constructed in the range of [0, 1]. If $P_{i}$ is greater than this random value, each onlooker bee will generate a new nectar position around the previous nectar source according to Equation (36). Meanwhile, the similar GEL was applied to determine the reserved nectar position. 

In this way, the iterative calculations were started. If $XP^{t}{}^{r}\;$ reaches the threshold $l_{im}$ corresponding to the pre-defined constraint condition, but employed bees fail to find better nectar positions, those nectar positions will be abandoned with the corresponding employed bees being transformed into onlooker bees. Subsequently, the onlooker bees will randomly generate new nectar sources in the search space to replace the abandoned ones. Here, the new nectar positions will be determined by employing the following equations until the optimal nectar position is outputted:

(39) $$xp_{i}^{t_{r}+1}=\{\begin{array}{ll} L_{i}+\mathrm{rand}\left(0,1\right)\left(U_{i}-L_{i}\right) & ,t_{r}\geq l_{\mathrm{im}}\\ xp_{i}^{t_{r}} & ,t_{r}<l_{\mathrm{im}} \end{array}$$

**Figure 3:**
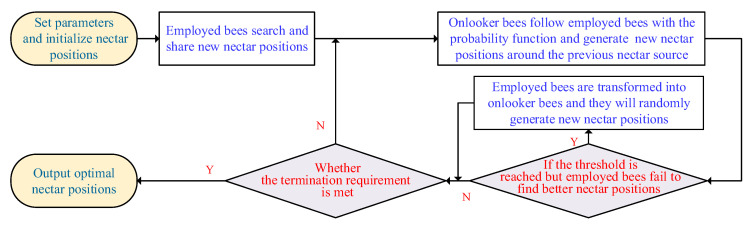
A flowchart of the ABC algorithm.

Finally, determine whether the ABC algorithm meets the termination requirement. If this is true, the interactive computations will be fulfilled, and the corresponding trade-off Pareto-optimal solutions will be found. Otherwise, new nectar spots will be generated, and the interactive computations will be continued. To better illustrate the iterative principle, Figure 3 gives the corresponding flowchart of the ABC algorithm.

### 3.3. Validation of Optimal Algorithm

Here, an estimation function was utilized to validate the effectiveness of the ABC algorithm, which has the following form:

(40) f(x,  y)=−xsin(4πx)+ysin(4πy+π)-1                      x,y∈[−2, 2]

**Figure 4:**
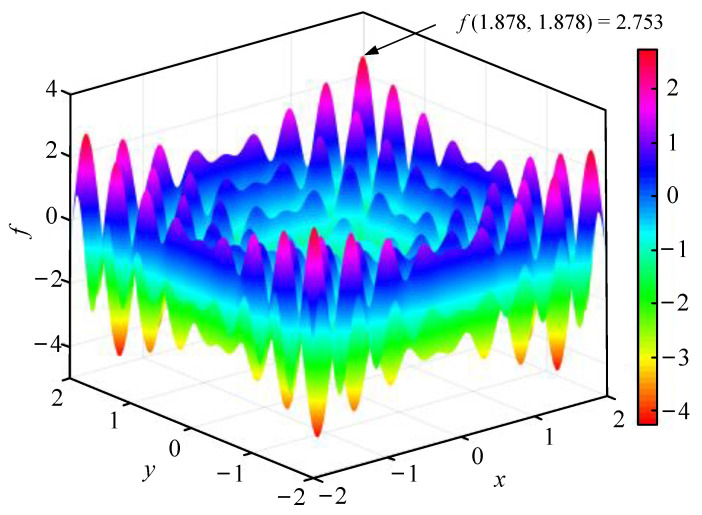
A surface colormap of an estimation function with x,  y∈[−2, 2].

Figure 4 illustrates the surface colormap of f(x,y) with x,y∈[−2, 2], which contains many local maximum points but only a global maximum point, i.e., $f_{\max}=2.753$ with x=y=1.878.

**Figure 5:**
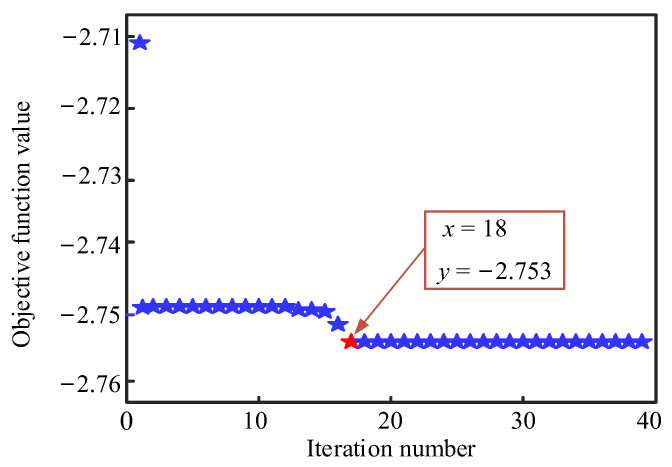
Objective function values calculated for the minimum optimization of $f_{0}(x,y)$ with different iteration numbers.

The ABC algorithm was used to carry out iterative calculations with the goal of achieving the smallest optimization result of $f_{0}(x,y)$, where $f_{0}(x,y)=-f(x,y)$. Figure 5 displays the corresponding objective function values with different iteration numbers in the optimization process. One can find that the iteration result converges quickly to the minimal value of −2.753 from the original value as the iteration number increases, which takes about 8.1 s, with a maximum iteration number of 18. Therefore, this algorithm can be utilized in the optimization design of bending and vibration resistant properties of the MECFRPSSs. It should be noted that since the effectiveness of the ABC algorithm was already confirmed in many studies [54,55,56,57], it was employed directly in the single-objective or multi-objective optimization design process.

## 4. Optimization Analysis

### 4.1. Single-Objective Optimization 

**Figure 6:**
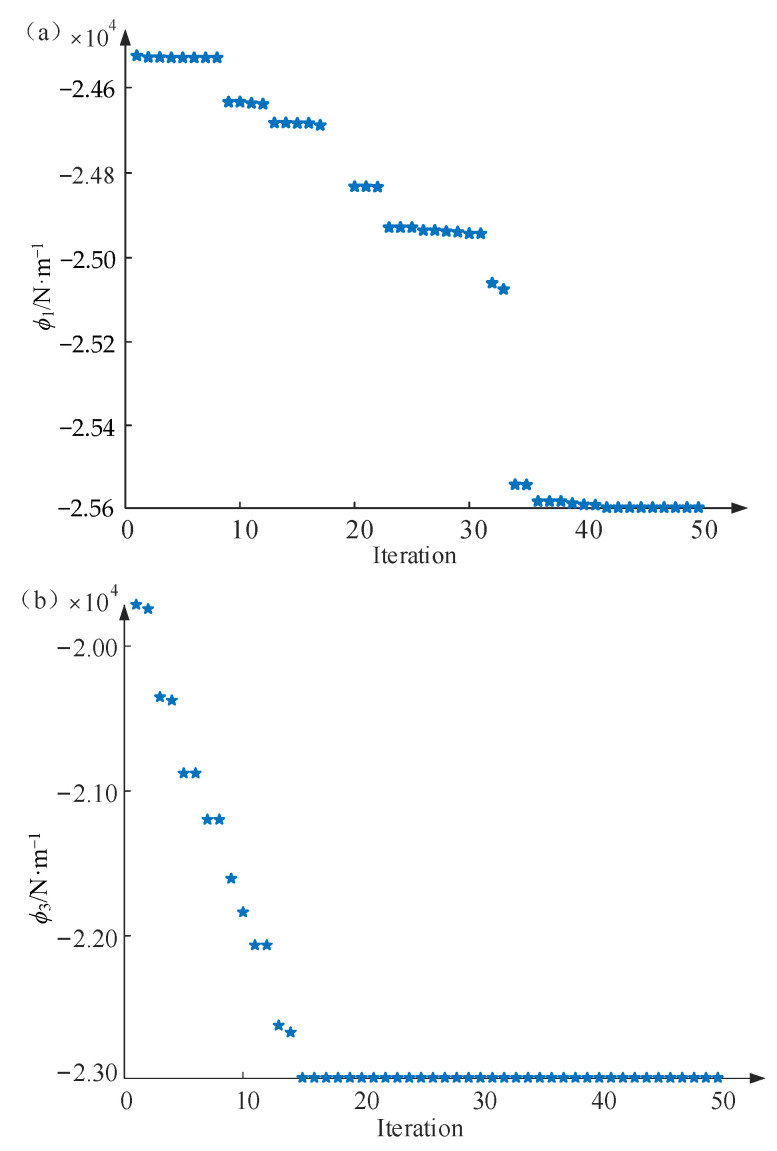
The calculated results of the MECFRPSS structure with different iteration numbers related to (**a**) bending and (**b**) vibration resistances.

Using the ABC algorithm described in Section 3, the optimization design of bending or vibration resistance of the MECFRPSS structure was performed using a single-objective function $\phi_{1}$ and $\phi_{3}$. Figure 6 illustrates the calculated results of the structure with different iteration numbers related to $\phi_{1}$ and $\phi_{3}$. In the iterative calculations, the input parameters of the ABC algorithm in Table 6 were employed. Additionally, the corresponding optimization results are provided in Table 7 and Table 8. It is worth mentioning that the mode order *N* was set as 3, and the first three weighting coefficients for dynamic stiffness were set as 0.7, 0.2, and 0.1, respectively.

It can be observed from Figure 6 that as the iteration number increased, the optimization results of bending and vibration resistances eventually became stable, regardless of the initial value chosen in the ABC algorithm. Thus, adopting the ABC algorithm can help the model to obtain the concerned optimal design variables, e.g., the geometric and material parameters (such as *h_s_* and *ρ_s_*), with high efficiency. In addition, based on the optimization analysis results in Table 7 and Table 8, it was not difficult to understand the physical mechanism behind the above optimization findings. Because a small value of $E_{s}$ and an appropriate value of $h_{s}$ all contribute to a high stiffness property of the structure studied, this will further lead to good bending and vibration suppression performance of the MECFRPSS structure.

### 4.2. Two-Objective Optimization

**Figure 7:**
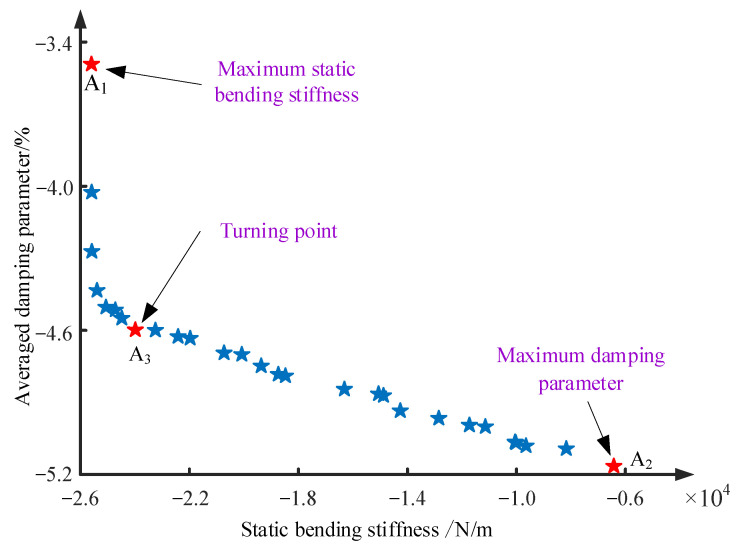
Pareto-optimal front when the two-objective optimization of the MECFRPSS structure is considered.

Furthermore, optimization calculation with two-objective functions was performed on the MECFRPSS structure based on the iterative calculation process of the ABC algorithm described in Section 3. Here, the objective functions include the maximum values of static bending stiffness and averaged damping parameters linked to the first three modes. Meanwhile, the thickness, modulus, and density ratios of MRE to panels were taken as the design variables. Figure 7 displays the Pareto-optimal front in which the corresponding critical points were also marked. Meanwhile, the final optimal design variables related to different critical points in this iterative calculation process are given in Table 9. 

From the results in Figure 7 and Table 9, it was found out that at the points A_1_ and A_2_, the static bending stiffness and the averaged damping parameter reached their respective extremes. So, when the two objective functions $\phi_{1}$ and $\phi_{2}$ were taken into account, a compromise design variable must be considered. That is the reason why the optimal thickness, modulus, and density results associated with the turning point A_3_ were between the related counterparts related to the points A_2_ and A_1._ As a result, according to the principle of Pareto-optimal solutions, it was suggested to adopt the optimal design variables that were closely related to point A_3._ In this way, the MECFRPSS structure will possess the optimal bending-vibration resistance.

### 4.3. Multi-Objective Optimization

**Figure 8:**
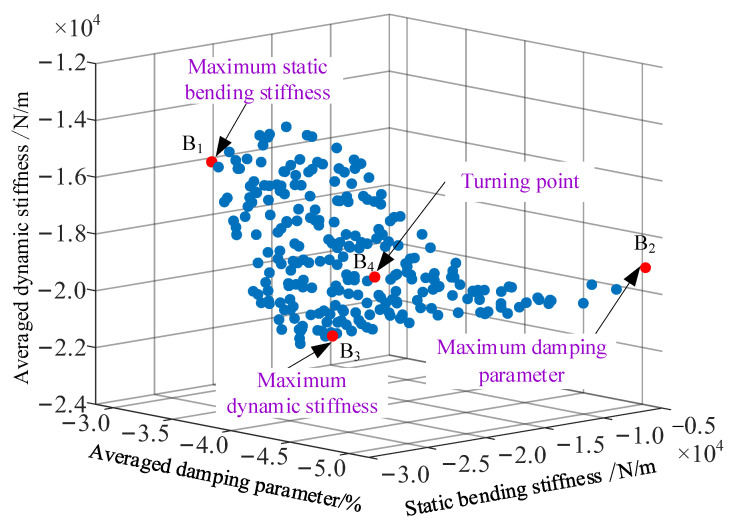
Pareto-optimal solutions when the multi-objective optimization of the MECFRPSS structure is considered.

Here, by taking the minimum values of static bending stiffness, averaged damping and dynamic stiffness parameters linked to the first three modes as the objective functions, the multi-objective optimization was undertaken on the MECFRPSS structure. Note that the same geometric and material parameters, design variables, and iteration parameters of the ABC algorithm listed in Table 6 were used in the iterative process. Figure 8 illustrates the corresponding Pareto-optimal solutions in this iterative calculation process, in which the corresponding critical points were also marked. Meanwhile, the final optimal design variables related to different critical points are shown in Table 10. 

The observation of Figure 8 and Table 10 indicate that the static bending stiffness was the largest at point B_1_, but the averaged dynamic stiffness was the smallest. At point B_2_, the damping parameter of the structure studied was the largest, but the static bending stiffness was the smallest. In addition, the averaged dynamic stiffness was the largest at point B_3_, but the structure did not have the best bending resistance and damping effect. Hence, it was difficult to find a perfectly optimal solution at the points B_1_, B_2_, and B_3_. To achieve the optimal bending and vibration suppression performances of the structure, a compromise decision must be made. Here, complying with the Pareto-optimal distribution, the optimal design variable results at the turning point B_4_ were suggested for such a structure in further engineering applications.

## 5. Conclusions

In this paper, the optimization design of bending and vibration resistances of the MECFRPSSs was conducted using the ABC algorithm, in which the thickness, modulus, and density ratios of MRE to panels were taken as the design variables with single-objective, dual-objective, and multi-objective optimizations being considered. Based on the optimization analysis results, it was proved that the ABC algorithm adopted in this study was reliable for performing the optimization design of bending- and vibration-resistant properties of the MECFRPSSs. When two-objective optimization was analysed with consideration of static bending stiffness and averaged damping parameters as objective functions, it was difficult to improve bending resistance and damping performance of the structure simultaneously as the values of three design variables increased continuously. For instance, increasing the thickness ratio only lead to the improvement of static bending stiffness rather than damping property. Therefore, it is recommended to choose the optimal design variables that are closely related to point A_3_ to obtain the optimum bending-vibration resistance. In addition, when multi-objective optimization work was finished, in which the static bending stiffness and averaged damping and dynamic stiffness parameters were taken as the objective functions, it was found that some compromise results of design variables need to be determined, which should also comply with the Pareto-optimal solutions. As a result, to better exert the bending-vibration suppression performance of the MECFRPSSs, the optimal design variables at the turning point B_4_ are suggested for such structures in further engineering applications.

## Figures and Tables

**Table 1:** Material and geometric parameters of the MECFRPSSs specimens.

Type	Value /mm	Type	Value /GPa	Type	Value/GPa	Type	Value	Type	Value/kg/m^3^
$L_{g}$	200	$E_{1}^{f}$	115	$E_{C2}$	7.8	$\eta_{C1}$	0.15	$\rho_{f}$	1370
$W_{d}$	80	$E_{1}^{w}$	163	$G_{12}^{f}$	7.1	$\eta_{C2}$	0.15		
$h_{a}$	0.60	$E_{\mathrm{IM}}$	72	$G_{12}^{w}$	47.5	$\eta_{C12}$	0.15		
$h_{m}$	0.30	$E_{C1}$	7.8	$G_{C12}$	4.5	$\eta_{C23}$	0.15	$\rho_{M}$	3300
$h_{M}$	2.00	$E_{2}^{f}$	9.5	$G_{C23}$	2.8	$v_{12}^{f}$	0.32		
$h_{w}$	0.31	$E_{2}^{w}$	143			$v_{12}^{w}$	0.35		

**Table 2:** Magnetic coefficients of MRE material.

Type	Value	Type	Value	Type	Value	Type	Value
$d_{1}$	2.564 × 10^1^	$e_{1}$	1.838	$f_{1}$	8.508 × 10^−1^	$g_{1}$	1.769 × 10^−1^
$d_{2}$	1.284 × 10^−1^	$e_{2}$	3.163	$f_{2}$	8.508 × 10^−1^	$g_{2}$	1.769 × 10^−1^
$d_{3}$	5.196	$e_{3}$	2.305	$f_{3}$	7.217 × 10^−1^	$g_{3}$	3.269 × 10^−1^
$d_{4}$	8.807 × 10^−4^	$e_{4}$	2.926 × 10^−1^	$f_{4}$	2.454 × 10^−8^	$g_{4}$	3.365 × 10^−1^

**Table 3:** Comparison of dynamic stiffness values calculated by the current model and Ref. [10] subjected to different magnetic induction amplitudes.

Magnetic Induction Intensity/mT	Mode	Present/N/m	Ref. [10]/N/m	Relative Deviation/%
0	1st	5322	5150	3.3
	2nd	27,820	27,760	0.2
16	1st	6279	6220	0.9
	2nd	30,420	29,670	2.5
35	1st	6900	6590	4.7
	2nd	31,110	30,840	0.9
62	1st	7276	7321	0.6
	2nd	31,540	32,200	2.0

**Table 4:** Comparison of damping ratios calculated by the current model and Ref. [10] subjected to different magnetic induction amplitudes.

Magnetic Induction Intensity/mT	Mode	Present/%	Ref. [10]/%	Relative Deviation/%
0	1st	5.98	6.50	8.0
	2nd	6.67	7.27	8.3
16	1st	5.99	6.53	8.3
	2nd	7.07	7.67	7.8
35	1st	6.03	6.58	8.4
	2nd	7.39	8.12	9.0
62	1st	6.05	6.62	8.6
	2nd	7.76	8.47	8.4

**Table 5:** Comparison of the maximum deformations and static bending stiffness values of the MECFRPSS structure calculated by ANSYS workbench software and the present model.

Force/N	Present *d*_max_/mm	ANSYS *d*_max_/mm	Relative Deviation/%	Present $\bar{K}$/N/m	ANSYS $\bar{K}$/N/m	Relative Deviation/%
20	0.686	0.656	−4.6	29,154.5	30,487.8	4.4
30	1.082	1.049	−3.1	27,726.4	28,598.7	3.0
40	1.536	1.487	−3.3	26,041.7	26,899.8	3.2

**Table 6:** Iteration parameters of the ABC algorithm.

Iteration Parameter	Value
Size of population	50~300
Maximum iteration number	50
Acceleration coefficient upper bound	1
Abandonment limit parameter	150

**Table 7:** Optimization results of bending resistant performance of the MECFRPSS structure.

Iteration	*ϕ*_1_	*h* *_s_*	*E_s_* × 10^−5^	*ρ* *_s_*
1	−24,527	0.72	6.27	2.51
5	−24,531	0.72	6.24	2.51
10	−24,635	0.74	6.16	2.51
25	−24,956	0.78	6.06	2.52
50	−25,599	0.79	5.89	2.52

**Table 8:** Optimization results of vibration resistant performance of the MECFRPSS structure.

Iteration	*ϕ*_3_	*h* *_s_*	*E_s_* × 10^−5^	*ρ* *_s_*
1	−19,845	0.28	6.26	2.95
5	−20,921	0.28	6.25	2.96
10	−21,802	0.29	6.17	2.95
25	−23,053	0.31	5.44	2.94
50	−23,054	0.31	5.44	2.95

**Table 9:** Optimal design variables related to different critical points in the two-objective optimization of the MECFRPSS structure.

Type	*h* *_s_*	*E_s_* × 10^−5^	*ρ* *_s_*	Equivalent Damping Parameter/%	Static Bending Stiffness /N∙m^−1^
A_1_	0.79	5.89	2.52	3.50	2.56 × 10^4^
A_2_	0.21	38.24	2.46	5.17	0.64 × 10^4^
A_3_	0.75	38.71	2.48	4.60	2.40 × 10^4^

**Table 10:** Optimal design variables related to different critical points in the multi-objective optimization of the MECFRPSS structure.

Type	*h* *_s_*	*E_s_* × 10^−5^	*ρ* *_s_*	Equivalent Damping Parameter/%	Equivalent Dynamic Stiffness/N∙m^−1^	Static Bending Stiffness/N∙m^−1^
B_1_	0.79	5.29	2.48	3.40	1.53 × 10^4^	2.56 × 10^4^
B_2_	0.21	38.24	2.64	5.17	1.90 × 10^4^	0.64 × 10^4^
B_3_	0.31	5.34	2.99	2.85	2.30 × 10^4^	0.98 × 10^4^
B_4_	0.62	14.61	2.58	4.20	1.92 × 10^4^	1.97 × 10^4^

## Data Availability

The data presented in this study are available on request from the corresponding author.

## References

[1] Hegde S., Shenoy B.S., Chethan K.N. (2019). Review on carbon fiber reinforced polymer (CFRP) and their mechanical performance. Mater. Today.

[2] Proença M., Garrido M., Correia J., Gomes M. (2021). Fire resistance behaviour of GFRP-polyurethane composite sandwich panels for building floors. Compos. Part B-Eng..

[3] Zheng H., Zhang W., Li B., Zhu J., Wang C., Song G., Wu G., Yang X., Huang Y., Ma L. (2022). Recent advances of interphases in carbon fiber-reinforced polymer composites: A review. Compos. B Eng..

[4] Nikbakt S., Kamarian S., Shakeri M. (2018). A review on optimization of composite structures Part I: Laminated composites. Compos. Struct..

[5] Li H., Gao Z.J., Zhao J., Ma H., Han Q.K., Liu J.G. (2021). Vibration suppression effect of porous graphene platelet coating on fiber reinforced polymer composite plate with viscoelastic damping boundary conditions resting on viscoelastic foundation. Eng. Struct..

[6] Ahmed O., Wang X., Tran M.-V., Ismadi M.-Z. (2021). Advancements in fiber-reinforced polymer composite materials damage detection methods: Towards achieving energy-efficient SHM systems. Compos. B Eng..

[7] Lee K.H., Park J.E., Kim Y.K. (2019). Design of a stiffness variable flexible coupling using magnetorheological elastomer for torsional vibration reduction. J. Intel. Mat. Syst. Str..

[8] Li H., Wang W.Y., Wang X.T., Han Q.K., Liu J.G., Qin Z.Y., Xiong J., Guan Z.W. (2020). A nonlinear analytical model of composite plate structure with an MRE function layer considering internal magnetic and temperature fields. Compos. Sci. Technol..

[9] Li H., Wang X.T., Hu X.Y., Xiong J., Han Q.K., Wang X.P., Guan Z.W. (2021). Vibration and damping study of multifunctional grille composite sandwich plates with an IMAS design approach. Compos. B Eng..

[10] Li H., Wang W.Y., Wang Q.S., Han Q.K., Liu J.G., Qin Z.Y., Xiong J., Wang X.P. (2022). Static and dynamic performances of sandwich plates with magnetorheological elastomer core: Theoretical and experimental studies. J. Sandw. Struct. Mater..

[11] Sun Q., Zhou J.X., Zhang L. (2003). An adaptive beam model and dynamic characteristics of magnetorheological materials. J. Sound Vib..

[12] Ramesh B.V., Vasudevan R., Kumar N.B. (2014). Vibration analysis of a laminated composite magnetorheological elastomer sandwich beam. Appl. Mech. Mater..

[13] Aguib S., Nour A., Zahloul H., Bossis G., Chevalier Y., Lançon P. (2014). Dynamic behavior analysis of a magnetorheological elastomer sandwich plate. Int. J. Mech. Sci..

[14] Babu V.R., Vasudevan R. (2016). Dynamic analysis of tapered laminated composite magnetorheological elastomer (MRE) sandwich plates. Smart Mater. Struct..

[15] Kozlowska J., Boczkowska A., Czulak A., Przybyszewski B., Holeczek K., Stanik R., Gude M. (2016). Novel MRE/CFRP sandwich structures for adaptive vibration control. Smart Mater. Struct..

[16] Aguib S., Nour A., Benkoussas B., Tawfiq I., Djedid T., Chikh N. (2016). Numerical simulation of the nonlinear static behavior of composite sandwich beams with a magnetorheological elastomer core. Compos. Struct..

[17] Settet A.T., Aguib S., Nour A., Zerrouni N. (2019). Study and analysis of the magneto-mechanical behavior of smart composite sandwich beam in elastomer. Mechanika.

[18] Eloy F.D.S., Gomes G.F., Ancelotti A.C., da Cunha S.S., Bombard A.J.F., Junqueira D.M. (2018). Experimental dynamic analysis of composite sandwich beams with magnetorheological honeycomb core. Eng. Struct..

[19] Eloy F., Gomes G.F., Ancelotti A.C., da Cunha S.S., Bombard A.J., Junqueira D.M. (2019). A numerical-experimental dynamic analysis of composite sandwich beam with magnetorheological elastomer honeycomb core. Compos. Struct..

[20] Zhang Y., Jin G., Chen M., Ye T., Yang C., Yin Y. (2020). Free vibration and damping analysis of porous functionally graded sandwich plates with a viscoelastic core. Compos. Struct..

[21] Garbowski T., Gajewski T., Grabski J.K. (2020). Role of transverse shear modulus in the performance of corrugated materials. Materials.

[22] Garbowski T., Gajewski T., Grabski J.K. (2020). Torsional and transversal stiffness of orthotropic sandwich panels. Materials.

[23] Staszak N., Gajewski T., Garbowski T. (2022). Shell-to-Beam Numerical Homogenization of 3D Thin-Walled Perforated Beams. Materials.

[24] Theulen J.C.M., Peijs A. (1991). Optimization of the bending stiffness and strength of composite sandwich panels. Compos. Struct..

[25] Liu T., Deng Z.C., Lu T.J. (2006). Design optimization of truss-cored sandwiches with homogenization. Int. J. Solids Struct..

[26] Li X., Li G., Wang C.H., You M. (2012). Optimum design of composite sandwich structures subjected to combined torsion and bending loads. Appl. Compos. Mater..

[27] Catapano A., Montemurro M. (2014). A multi-scale approach for the optimum design of sandwich plates with honeycomb core Part II: The optimization strategy. Compos. Struct..

[28] Hao J., Wu X., Oporto G., Liu W., Wang J. (2020). Structural analysis and strength-to-weight optimization of wood-based sandwich composite with honeycomb core under three-point flexural test. Eur. J. Wood Wood Prod..

[29] Uzay C., Acer D.C., Geren N. (2022). A method for the optimal design of low-density polymer foam core sandwiches using FEA and multi objective optimization of design variables. J. Polym. Eng..

[30] Karakaya S., Soykasap O. (2011). Natural frequency and buckling optimization of laminated hybrid composite plates using genetic algorithm and simulated annealing. Struct. Multidiscipl. Optim..

[31] Honda S., Narita Y. (2011). Vibration design of laminated fibrous composite plates with local anisotropy induced by short fibers and curvilinear fibers. Compos. Struct..

[32] Madeira J., Araújo A., Soares C.M., Ferreira A. (2015). Multiobjective design of viscoelastic laminated composite sandwich panels. Compos. B Eng..

[33] Alfouneh M., Ji J.C., Luo Q.T. (2020). Optimal design of multi-cellular cores for sandwich panels under harmonic excitation. Compos. Struct..

[34] Wang X., Li X., Yue Z.-S., Yu R.-P., Zhang Q.-C., Du S.-F., Yang Z.-K., Han B., Lu T.J. (2021). Optimal design of metallic corrugated sandwich panels with polyurea-metal laminate face sheets for simultaneous vibration attenuation and structural stiffness. Compos. Struct..

[35] Njim E.K., Bakhy S.H., Al-Waily M. (2021). Optimization design of vibration characterizations for functionally graded porous metal sandwich plate structure. Mater. Today.

[36] Tu T.M., Quoc T.H. (2010). Finite element modeling for bending and vibration analysis of laminated and sandwich composite plates based on higher-order theory. Comput. Mater. Sci..

[37] Cui X.Y., Liu G.R., Li G.Y. (2011). Bending and vibration responses of laminated composite plates using an edge-based smoothing technique. Eng. Anal. Bound. Elem..

[38] Natarajan S., Manickam G. (2012). Bending and vibration of functionally graded material sandwich plates using an accurate theory. Finite Elem. Anal. Des..

[39] Kapuria S., Nath J.K. (2013). On the accuracy of recent global–local theories for bending and vibration of laminated plates. Compos. Struct..

[40] Khiloun M., Bousahla A.A., Kaci A., Bessaim A., Tounsi A., Mahmoud S.R. (2020). Analytical modeling of bending and vibration of thick advanced composite plates using a four-variable quasi 3D HSDT. Eng. Comput..

[41] Urban F., Bo A., Middendorf P. (2021). Development and validation of a method for linear-viscoelastic characterization of the dynamic complex modulus of short-fiber reinforced plastics using flexural resonances. Polym. Test.

[42] Li H., Lv H.Y., Sun H., Qin Z.Y., Xiong J., Han Q.K., Liu J.G., Wang X.P. (2021). Nonlinear vibrations of fiber-reinforced composite cylindrical shells with bolt tube boundary conditions. J. Sound Vib..

[43] Jolly M.R., Carlson J.D., Munoz B.C. (1996). A model of the behaviour of magnetorheological materials. Smart Mater. Struct..

[44] Li H., Wu T.F., Gao Z.J., Wang X.T., Ma H., Han Q.K., Qin Z.Y. (2020). An iterative method for identification of temperature and amplitude dependent material parameters of fiber-reinforced polymer composites. Int. J. Mech. Sci..

[45] Ali A., Salem A.M.H., Muthalif A.G.A., Bin Ramli R., Julai S. (2022). Development of a performance-enhanced hybrid magnetorheological elastomer-fluid for semi-active vibration isolation: Static and dynamic experimental characterization. Materials.

[46] Li H., Li Z.L., Xiao Z.Y., Wang X.P., Xiong J., Zhou J., Guan Z.W. (2021). Development of an integrated model for prediction of impact and vibration response of hybrid fiber metal laminates with a viscoelastic layer. Int. J. Mech. Sci..

[47] Shcherbakov V.P., Tsyganov I.B., Dmitriev O.Y., Polyakova T.I. (2007). Theoretical principles of experimental determination of fibre flexural rigidity. Fibre. Chem..

[48] Li H., Xue P.C., Guan Z.W., Han Q.K., Wen B.C. (2018). A new nonlinear vibration model of fiber-reinforced composite thin plate with amplitude-dependent property. Nonlinear. Dyn..

[49] Ramian A., Jafari-Talookolaei R.-A., Valvo P.S., Abedi M. (2021). Free vibration analysis of a laminated composite sandwich plate with compressible core placed at the bottom of a tank filled with fluid. Structures.

[50] Shahgholian-Ghahfarokhi D., Rahimi G., Liaghat G., Degenhardt R., Franzoni F. (2020). Buckling prediction of composite lattice sandwich cylinders (CLSC) through the vibration correlation technique (VCT): Numerical assessment with experimental and analytical verification. Compos. Part B-Eng..

[51] Khan S.U., Li C.Y., Siddiqui N.A., Kim J.-K. (2011). Vibration damping characteristics of carbon fiber reinforced composites containing multi walled carbon nanotubes. Compos. Sci. Technol..

[52] Mukherjee S., Jafarali P., Prathap G. (2005). A variational basis for error analysis in finite element elastodynamic problems. J. Sound Vib..

[53] Sun W., Wang Z., Liu R., Yan X. (2017). Inverse identification of the frequency-dependent mechanical parameters of a viscoelastic core layer based on the vibration response. J. Appl. Sci..

[54] Apalak M.K., Karaboga D., Akay B. (2014). The artificial bee colony algorithm in layer optimization for the maximum fundamental frequency of symmetrical laminated composite plates. Eng. Optimiz..

[55] Aslan S. (2020). A comparative study between artificial bee colony (ABC) algorithm and its variants on big data optimization. Memet. Comput..

[56] Li G.Q., Niu P.F., Xiao X.J. (2012). A Development and investigation of efficient artificial bee colony algorithm for numerical function optimization. Appl. Soft Comput..

[57] Madeira J., Araújo A., Soares C.M. (2020). Multiobjective optimization for vibration reduction in composite plate structures using constrained layer damping. Comput. Struct..

